# Synthesis of the RTH-type layer: the first small-pore, two dimensional layered zeolite precursor†
†Electronic supplementary information (ESI) available: Figure of the RTH-type layer with T-site labels and log-plot high resolution argon adsorption isotherms. See DOI: 10.1039/c5sc02325d


**DOI:** 10.1039/c5sc02325d

**Published:** 2015-07-27

**Authors:** Joel E. Schmidt, Dan Xie, Mark E. Davis

**Affiliations:** a Chemical Engineering , California Institute of Technology , Pasadena , CA 91125 , USA . Email: mdavis@cheme.caltech.edu; b Chevron Energy Technology Company , Richmond , CA 94802 , USA

## Abstract

There are several distinct two-dimensional zeolite building layers that can condense through different stacking arrangements of the layers to form various three-dimensional framework materials. All known building layers are dense layers in that they do not contain 8-membered ring (MR) or larger pores perpendicular to the two-dimensional layers. Herein, we report a new material (CIT-10) that consists of a two-dimensional layer (termed “rth” layer) that contains an 8 MR perpendicular to the layer. Calcination of CIT-10 forms pure-silica **RTH** (SSZ-50). CIT-10 can be pillared to form a new framework material with a three-dimensional pore system of 8 and 10 MRs, denoted CIT-11, that can be calcined to form a new microporous material, denoted CIT-12.

## Abstract 2

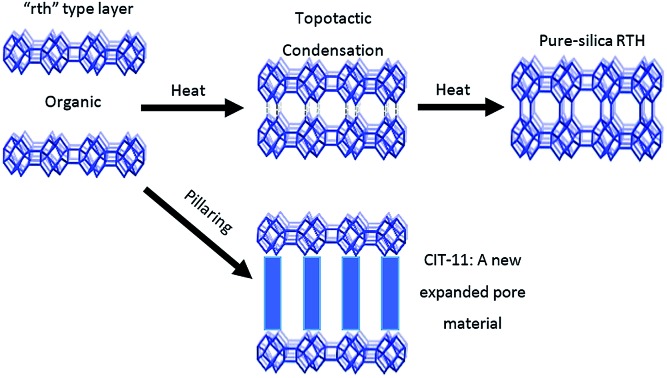
The “rth” type layer is the first porous 2D zeolite layer; it forms zeolite **RTH** via topotactic condensation or can be pillared to create a thermally stable, expanded pore material.

## 1. Introduction

The rate of discovery of new microporous materials has accelerated in recent years due to factors including new organic structure directing agents, the use of fluoride as a mineralizing agent as well as germanium as a heteroatom in syntheses.^1^–^6^ Much of this discovery is motivated by the fact that a single framework and composition are normally found to achieve optimal performance in a process.^7^ Another synthesis strategy that has received increased attention is that of synthesizing layered silicates that can directly form microporous materials *via* topotactic condensation, or can be pillared using silyating agents, forming structures with larger pores (generally larger by 2 tetrahedral atoms) than would be formed *via* topotactic condensation.^8^–^14^

In a topotactic condensation, a three-dimensional (3D) framework structure is formed from a two-dimensional (2D) layered silicate by condensation of surface silanol groups (Si-OH), releasing water. Some of the initial framework materials prepared *via* topotactic condensation were **FER**, formed from the layered precursor denoted PREFER,^15^ and **MWW**, formed from the layered precursor MCM-22(P).^16^ After these pioneering efforts, several additional frameworks have been prepared using topotactic condensation, and they include (layered precursor given in parentheses): **AST** (β-helix-layered silicate),^17^**CAS-NSI** intermediate (EU-19, NU-6(1)),^18^–^20^ **CDO** (PLS-1, RUB-36),^19^,^21^ **FER** (PREFER),^15^**MTF** (HPM-2),^22^**MWW** (MCM-22),^16^**PCR** (IPC-4 prepared by disassembly of **UTL**),^23^**RRO** (RUB-39),^24^**RWR** (RUB-18)^25^ and **SOD** (RUB-15).^26^ The layered materials that form 3D frameworks can be built by different stacking arrangements of a few 2D building layers (such as **CAS**, **FER**, **HEU**, **MWW** and **RWR**).^8^,^9^ Additionally, methods have been developed to prepare **MFI** nanosheets that are a single unit cell thick,^27^,^28^ however, this material generally is not considered to be a building layer.^29^

The silanol groups of the layered zeolite precursors can also be used to prepare larger pore materials through a pillaring process. This process normally uses dichlorodimethylsilane or diethoxydimethylsilane to react with the silanol groups to form pillars that are coordinated to two methyl groups (or two hydroxyl groups after calcination). This process is typically carried out in acidic media under hydrothermal conditions. Some of the layered materials that have been pillared include PREFER, MWW(P), PLS-1, MCM-47, RUB-36, RUB-39 and Nu-6(1).^8^,^30^,^31^ Additionally, related strategies to prepare porous materials include delamination or exfoliation. Recently, it has also been shown to be possible to introduce catalytic activity in the pillars.^32^,^33^ Excellent reviews of the above strategies are available.^8^,^9^

All of the previously reported layered zeolite precursors are dense layers, that is, they contain no pores (8-membered ring (MR) or larger) that are perpendicular to the layers. The **MWW** layer contains a sinusoidal 10 MR channel parallel to the *ab*-plane, but it is still dense as this channel is not perpendicular to the layer. However, the nanosheets of **MFI** that are single unit cell thick do contain a 10 MR perpendicular to the layer, a medium size pore.^27^ We recently reported a method to prepared high-silica heulandite (denoted CIT-8) *via* topotactic condensation from a layered precursor (denoted CIT-8P), that was prepared using a diquaternary organic structure directing agent (OSDA) in fluoride-mediated syntheses.^34^ In that case, the building layer that forms CIT-8 (**HEU**) is the same as that of RUB-41 (**RRO**), but is formed from a different stacking of the **HEU** building layer: AA stacking gives the **RRO** structure while AA′ stacking (where the A′-layer is related to the A-layer by a 180 degree rotation) gives the **HEU** structure.^25^ CIT-8 was prepared from fluoride-mediated, aluminosilicate inorganic conditions across a relatively narrow composition range. It is interesting to note with this material that the OSDA used was considerably larger than what is normally found in preparing topotactic materials such as piperdines,^15^ as well as methyl, ethyl and propyl substituted ammoniums.^31^

In the absence of aluminium, we have found conditions to prepare the layered precursor to pure-silica **RTH**, denoted CIT-10. This layered material can be directly calcined to prepare pure-silica **RTH** (SSZ-50 (ref. 35)) or can be pillared, leading to a new microporous material (denoted CIT-11), that is stable to calcination (calcined material denoted CIT-12). CIT-10 is a layered material composed of a new type of 2D building layer (termed “rth”, according to the nomenclature defined in ref. 9) containing an 8 MR channel going through the layer (8 MR pore dimensions of 5.6 × 2.5 Å). The discovery of CIT-10 adds a new group of layered zeolite precursors and is the first to contain an 8 MR through the 2D layer. Pure-silica **RTH** is now the sixth known microporous material that can be obtained by both direct synthesis and topotactic condensation.

## 2. Experimental

### 2.1. OSDA synthesis

**Fig. 1:**
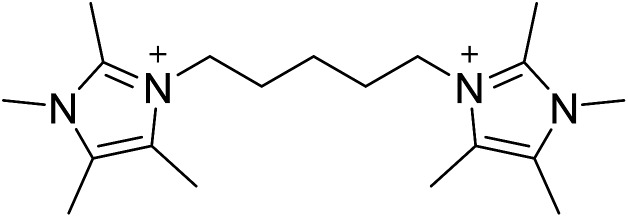
OSDA used to prepare CIT-10.

The diquaternary OSDA used in this work (shown in Fig. 1) was synthesized by reacting 200 mmol of 1,2,4,5-tetramethylimidazole (TCI Chemicals) with 100 mmol of 1,5-dibromopentane (Aldrich) at reflux in methanol overnight. The solvent was then removed using rotary evaporation and the product washed with ether. The product was verified using ^13^C NMR in D_2_O with methanol added as an internal standard. ^13^C-NMR (125 MHz, D_2_O): *δ* 7.76, 7.82, 9.61, 22.82, 28.58, 31.42, 44.72, 124.84, 126.03, 141.95. The product was ion exchanged to hydroxide form using Dowex Marathon A exchange resin and the final product concentration was determined using a Mettler-Toledo DL22 autotitrator using 0.01 M HCl as the titrant.

### 2.2. Synthesis of CIT-10

Tetraethylorthosilicate was added to the OSDA in its hydroxide form in a Teflon Parr Reactor. The container was closed and stirred overnight to allow for complete hydrolysis. The lid was then removed, and the ethanol and some water were allowed to evaporate under a stream of air. Once the gel was dry, a small amount of water was added to obtain a homogenous liquid. Then aqueous HF was added and the mixture was stirred by hand. A second evaporation step was then used to give a final gel molar ratio of 1SiO_2_ : 0.5R_1/2_(OH) : 0.5HF : 4H_2_O. Seeds of CIT-10 were then added and the autoclave was sealed and placed in a rotating oven (43 rpm) at 175 °C. Aliquots of the material were taken periodically by first quenching the reactor in water and then removing enough material for powder X-ray diffraction (PXRD). Synthesis times for pure silica **RTH** were on the order of 20 days when no seeds were added and 10 days when seeds were added. The product was recovered *via* centrifugation and was washed with water 3 times, a final time with acetone and dried in air at 100 °C.

### 2.3. Pure-silica **RTH**

The as-made material prepared in Section 2.2 was calcined in breathing grade air. The material was heated to 150 °C at 1 °C min^–1^, held for three hours, then heated to 580 °C at 1 °C min^–1^ and held for six hours to assure complete combustion of the organic.

### 2.4. Pillaring of CIT-10 to obtain CIT-11

The procedure that led to the pillared product with the highest crystallinity (judged using PXRD) was found to be as follows. 500 mg of CIT-10 were placed in a 45 mL Teflon Parr Reactor, then 20 g of a 1.25 M solution of HCl in ethanol were added. Finally 500 mg of silyating agent (dichlorodimethylsilane or diethoxydimethylsilane, both were found to produce a similar product) were added. The reactor was sealed and place in a rotating oven at 175 °C for 24 hours. The product was recovered *via* centrifugation and was washed one time with absolute ethanol, three times with water and finally one time with acetone and then dried in air at 100 °C.

### 2.5. CIT-12

CIT-12 was obtained by the calcination of CIT-11 using the procedure described in Section 2.3.

### 2.6. Characterizations

Liquid NMR spectra were recorded with a 500 MHz spectrometer. ^13^C and ^29^Si solid-state NMR were performed using a Bruker DSX-500 spectrometer (11.7 T) and a Bruker 4 mm MAS probe. The spectral operating frequencies were 500.2 MHz, 125.721 MHz and 99.325 MHz for ^1^H, ^13^C and ^29^Si nuclei, respectively. Spectra were referenced to external standards as follows: tetramethylsilane (TMS) for ^1^H and ^29^Si and adamantane for ^13^C as a secondary external standard relative to tetramethylsilane. Samples were spun at 8 kHz for ^13^C and ^29^Si MAS and CPMAS NMR experiments. Thermogravimetric analysis was performed on a Perkin-Elmer STA 6000 with a ramp of 1 °C min^–1^ to 900 °C under air atmosphere. Argon physical adsorption isotherms were performed at 87 K using a Quantachrome Autosorb iQ and were conducted using a quasi-equilibrium, volumetric technique.^36^ PXRD data were collected on a Rigaku MiniFlex II with Cu Kα radiation. Variable temperature Powder X-ray diffraction (PXRD) patterns were collected from 30 °C to 580 °C at increments of 50 °C under ambient conditions, using a PANalytical Empyrean powder diffractometer (Cu Kα radiation) equipped with an Anton Paar HTK 1200N high-temperature chamber. The sample was stabilized at each measurement temperature for 15 min before starting each measurement. The temperature ramp between two consecutive temperatures was 5 °C min^–1^. Scanning electron microscope (SEM) images were acquired on a ZEISS 1550 VP FESEM, equipped with in-lens SE. Energy-dispersive X-ray spectroscopy (EDS) spectra were acquired with an Oxford X-Max SDD X-ray Energy Dispersive Spectrometer system. Three-dimensional electron diffraction data were collected using the rotation electron diffraction (RED) technique.^37^,^38^ The RED software was installed on a JEOL 2010 microscope operating at 200 kV, and data were collected over a tilt range of ±50° with a tilt step of 0.50°, the exposure time is 3 seconds per tilt step.

## 3. Results and discussions

### 3.1. Synthesis of CIT-10 and calcination to produce pure-silica **RTH**

**Fig. 2:**
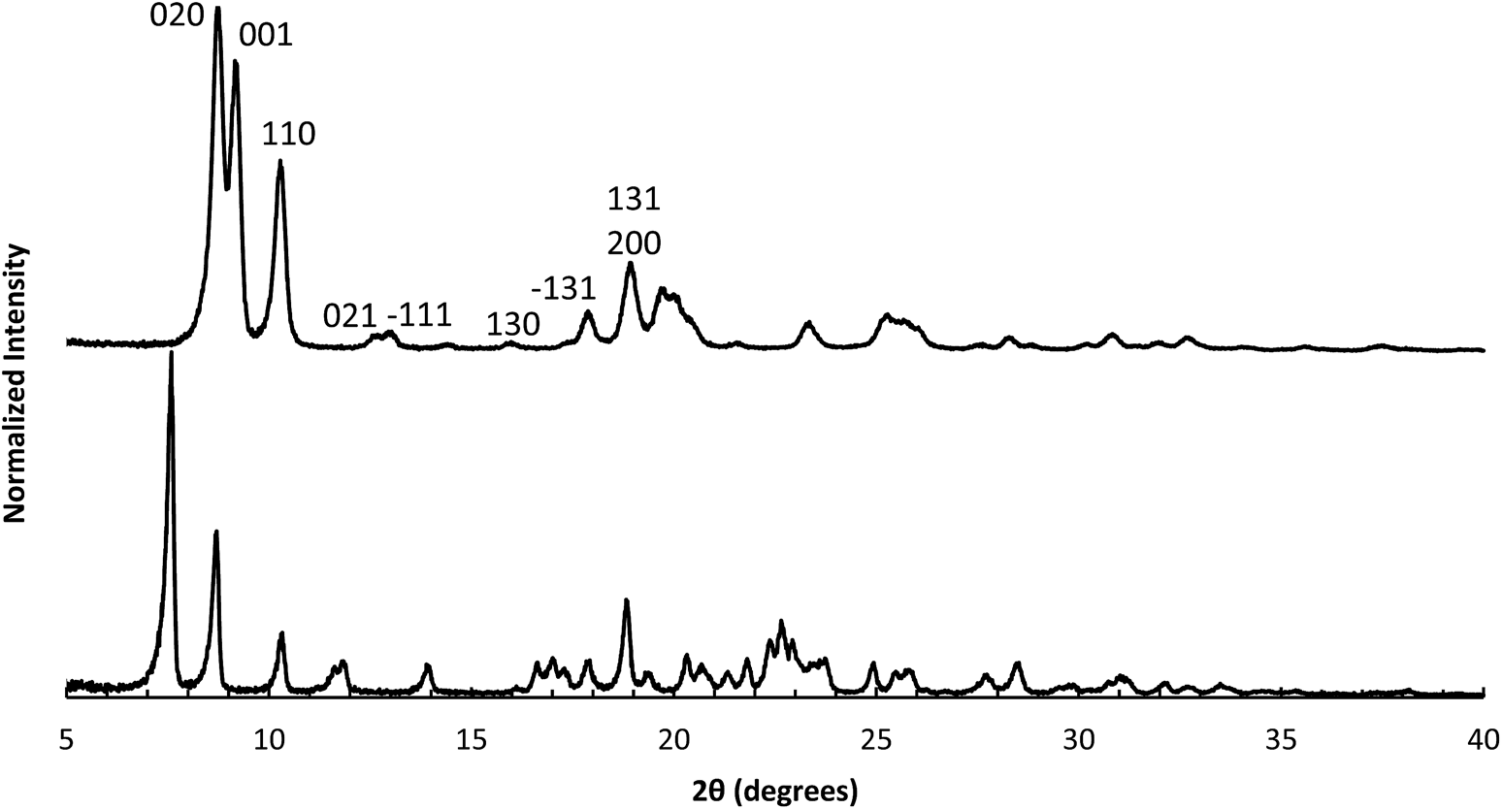
PXRD of CIT-10 (lower) and calcined CIT-10 (pure silica **RTH**, upper) with selected crystallographic indicies.

**Fig. 3:**
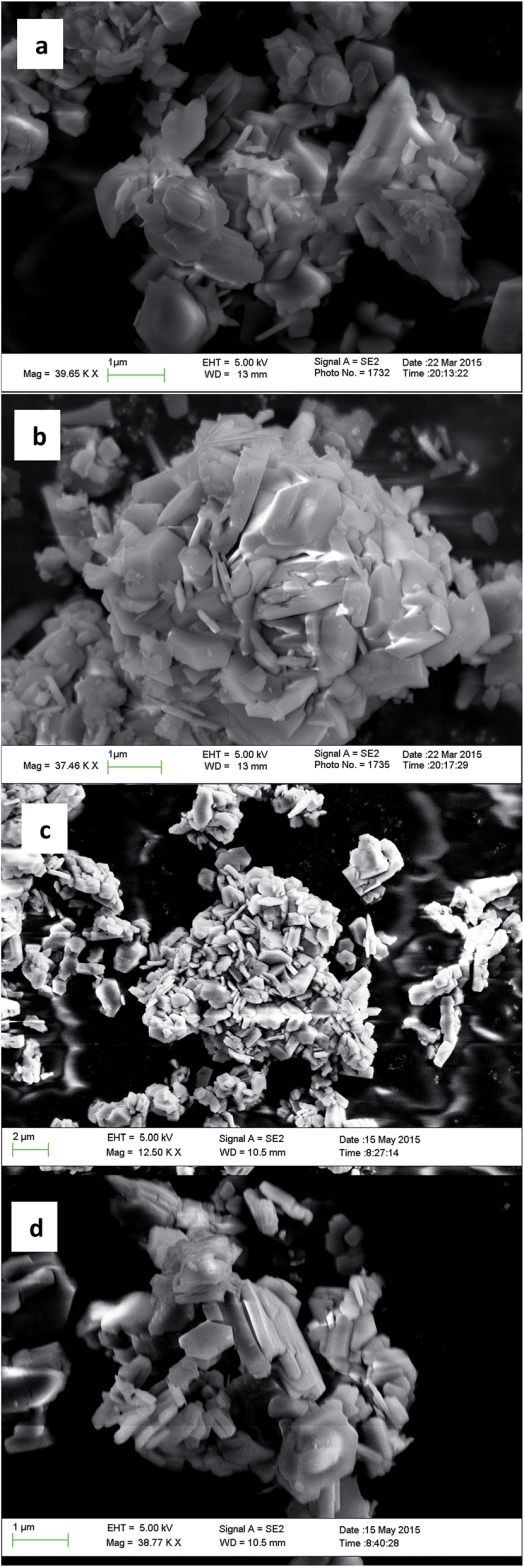
SEM images of (a) CIT-10, (b) Si-**RTH**, (c) CIT-11 and (d) CIT-12.

We have recently been investigating imidazolium OSDAs in the synthesis of microporous materials, and have found that they are able to produce a wide range of phases including **LTA**,^39^**RTH**,^40^,^41^ **STW**,^42^**CSV**^43^ and **HEU**^34^ in addition to a number of additional phases discussed in the previous references. While the majority of these products are microporous materials that were made with OSDAs intact inside the framework, the high-silica **HEU** (CIT-8) could be prepared from a layered precursor (CIT-8P). CIT-8P was synthesized in fluoride-media from a gel containing a relatively high amount of aluminium (gel Si/Al = 15 or 20). The result of finding a layered material in these conditions led us to continue to explore similar inorganic conditions. In aluminum-free syntheses, we have reported that diquats formed from tetramethylimidazole can be used to prepare pure-silica **CSV** (CIT-7).^43^ However, under similar conditions, we found that the diquat containing a five-carbon chain linker length led to a phase that could not be identified (shown in Fig. 2). Upon calcination, this material yielded a phase that was easily identified as pure-silica **RTH** (Fig. 2). This is the second reported method to synthesize pure-silica **RTH**, and may broaden its evaluation in applications as previously, the synthesis of this type of material required a difficult to prepare OSDA.^35^ SEM images of CIT-10 and pure-silica **RTH** are shown in Fig. 3. These images do not show solids with regular morphologies that are commonly observed in highly crystalline materials, but instead show morphologies resembling thick plates. Plate-like morphology is common in layered materials, however, the thickness of the plates in these samples is unusual.

**Fig. 4:**
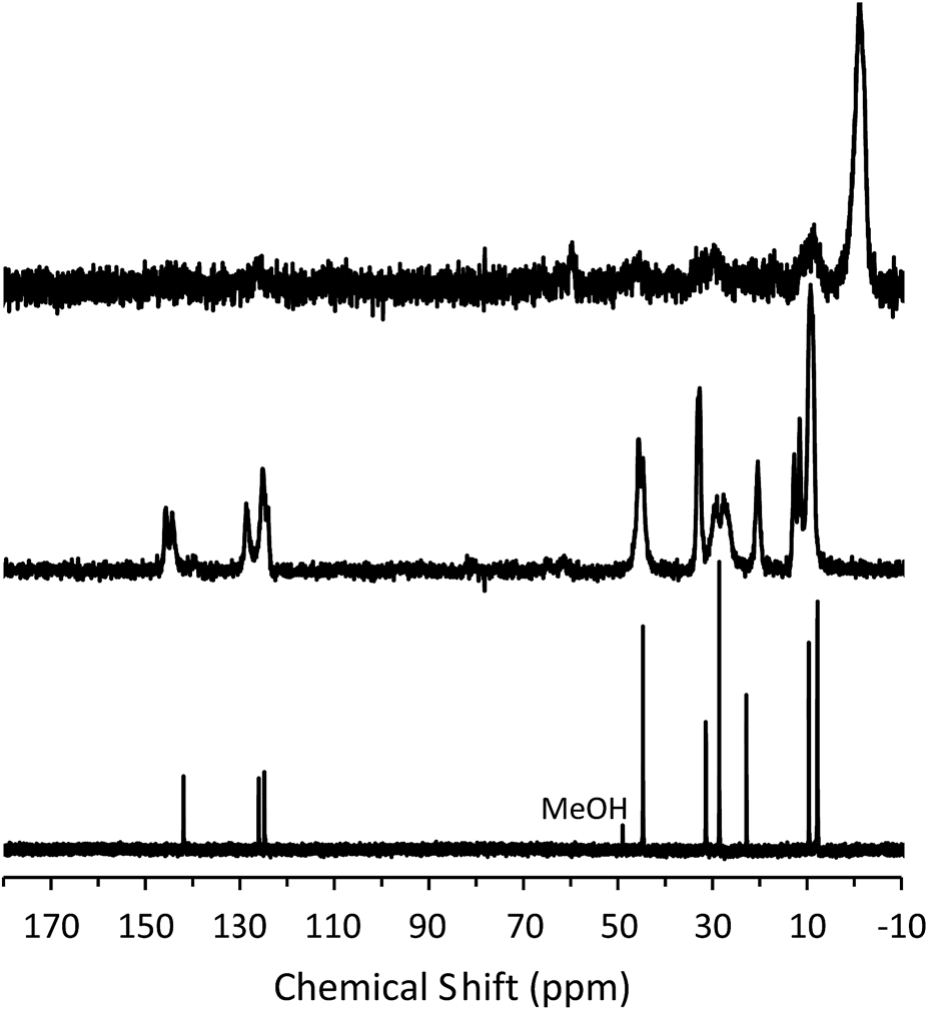
^13^C NMR of the diquat in D_2_O (lower, methanol added as an internal standard), ^13^C CPMAS NMR of CIT-10 showing the occluded diquat (middle) and ^13^C CPMAS NMR of CIT-11 (upper).

**Fig. 5:**
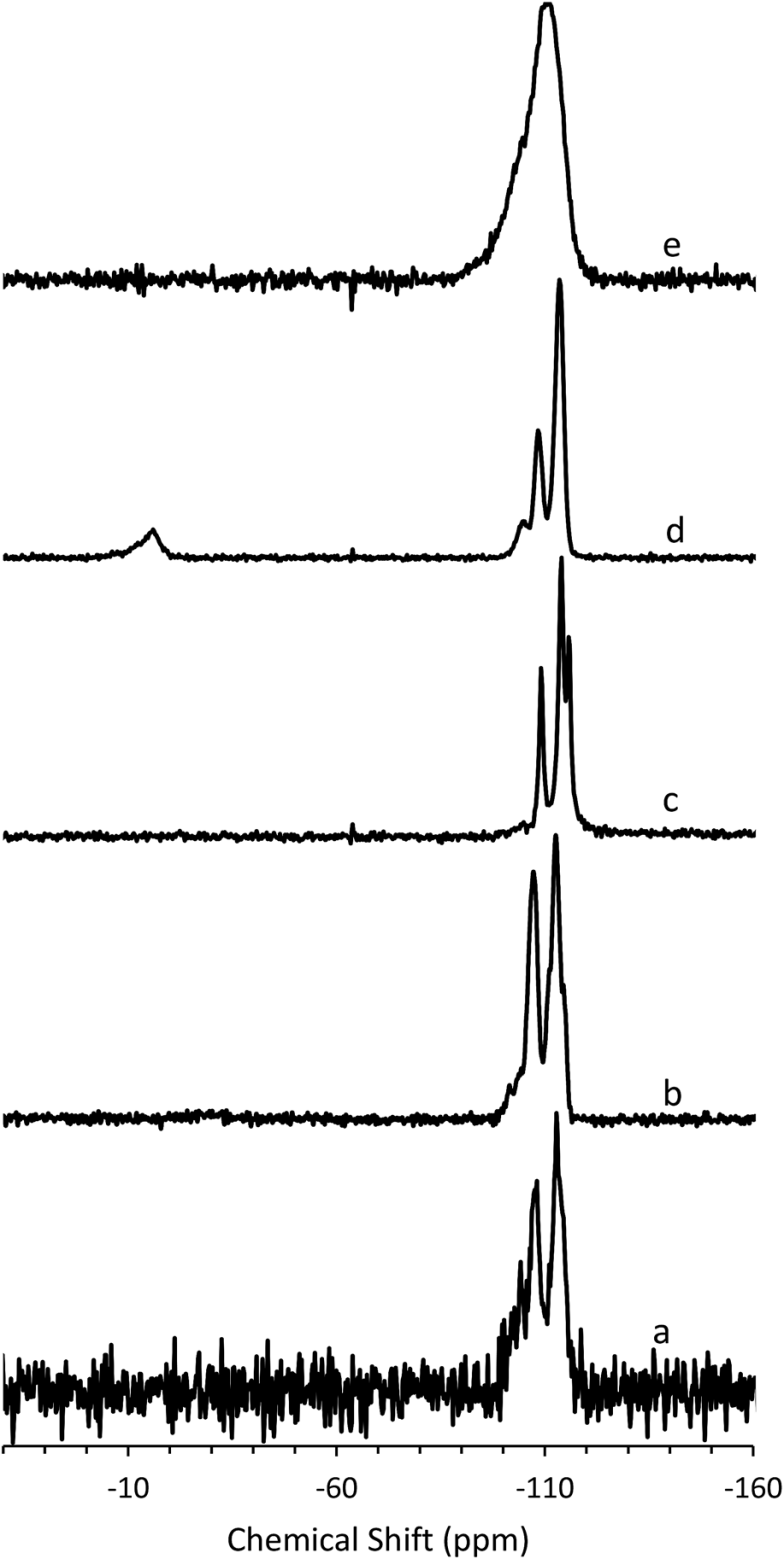
^29^Si (a) MAS NMR of CIT-10, (b) CPMAS NMR of CIT-10, (c) MAS NMR of pure-silica **RTH** prepared by calcination of CIT-10, (d) MAS NMR of CIT-11 and (e) MAS NMR of CIT-12.

To determine the mechanism of formation of pure-silica **RTH**, the materials were studied using ^13^C CPMAS NMR, ^29^Si MAS and CPMAS NMR and variable temperature PXRD. The ^13^C CPMAS NMR of CIT-10 (Fig. 4) shows that the diquat OSDA was occluded intact in the material. It is interesting to note that many of the peaks in this spectrum are split, indicating that otherwise equivalent carbon atoms are present in non-equivalent environments; this has been previously reported in layered materials.^10^ The ^29^Si MAS and CPMAS NMRs of CIT-10 are shown in Fig. 5. CIT-10 was studied using CPMAS NMR in addition to MAS NMR to confirm the resonances (organic-containing materials often exhibit a poor signal-to-noise ratio). In the as-made material there are three resonances at –113, –107 and –102 ppm with approximate area ratios of 8 : 5 : 3. The signals at –113 and –107 ppm are assigned to Q^4^ silicon, Si(OSi)_4_ coordination, while the signal at –102 ppm is assigned to Q^3^ silicon, Si(OSi)_3_(OH) coordination. The presence of Q^3^ silicon is expected in a layered material. The ratio of Q^3^/(Q^3^ + Q^4^) silicon in the as made material is 0.23, which is very close to the theoretical value of 0.25 (see the ESI Fig. S1† for additional details). Upon calcination, the ^29^Si MAS NMR spectrum no longer shows the presence of any Q^3^ silicon and instead shows 3 resonances in the Q^4^ region at –116, –114 and –109 ppm, with area ratios of 1 : 2 : 1. These area ratios agree with the crystal structure of **RTH**, as it contains 4 independent T-sites.

**Fig. 6:**
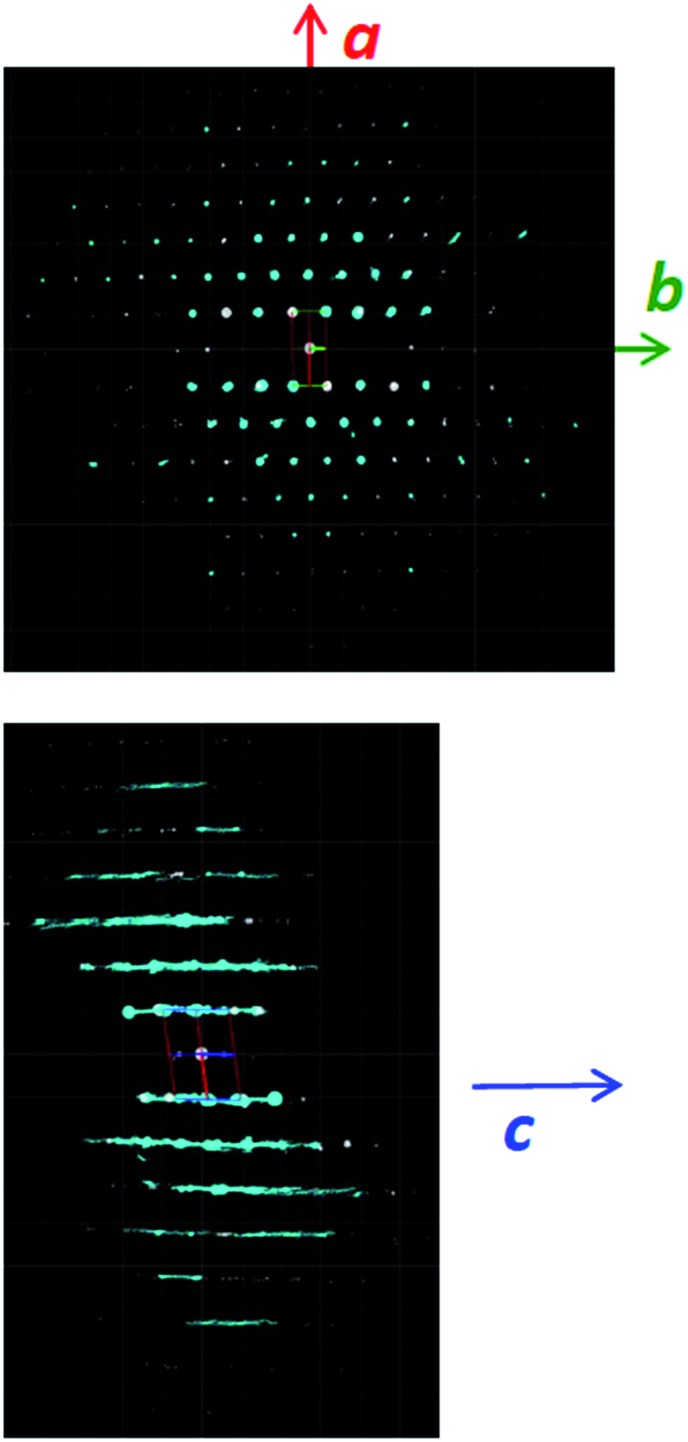
RED structure analysis of CIT-10.

**Fig. 7:**
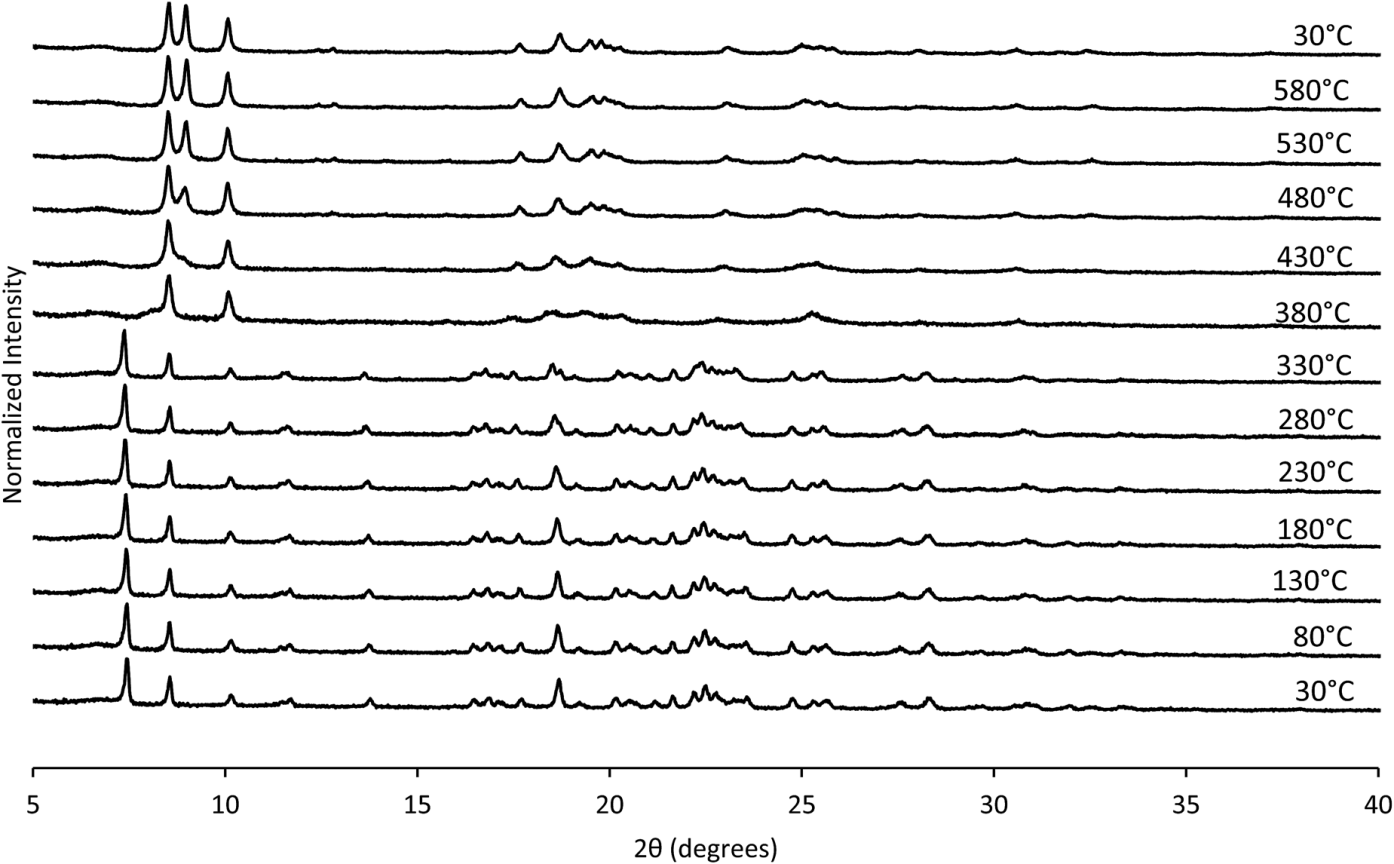
Variable temperature PXRD of CIT-10.

The structural mechanism of condensation was determined by using variable temperature PXRD as well as RED. The variable temperature PXRD of CIT-10 is shown in Fig. 7. When compared with the PXRD patterns of **RTH** in Fig. 2 (labelled with the crystallographic indices), it is apparent that peak positions for *hk*0 reflections remain during heating, while the peak positions for the *hkl* (*l* ≠ 0) reflections are shifted to higher 2*θ* angles (*i.e.*, lower *d*-spacing). This result indicates that the 3D **RTH** structure forms *via* topotactic condensation along the *c*-axis, and that the *a* and *b* axes are intact in the layered material. The structural change was further confirmed by studying CIT-10 using RED (Fig. 6). The RED clearly shows that CIT-10 is ordered in the *a* and *b* directions (indicated by clearly defined diffraction spots), but that some disorder is present in the *c* direction (indicated by diffraction streaks between diffraction spots). Thus, results from using both techniques confirm that CIT-10 contains 2D sheets in the *a* and *b* directions that are separated by a disordered organic in the *c* direction.

**Fig. 8:**
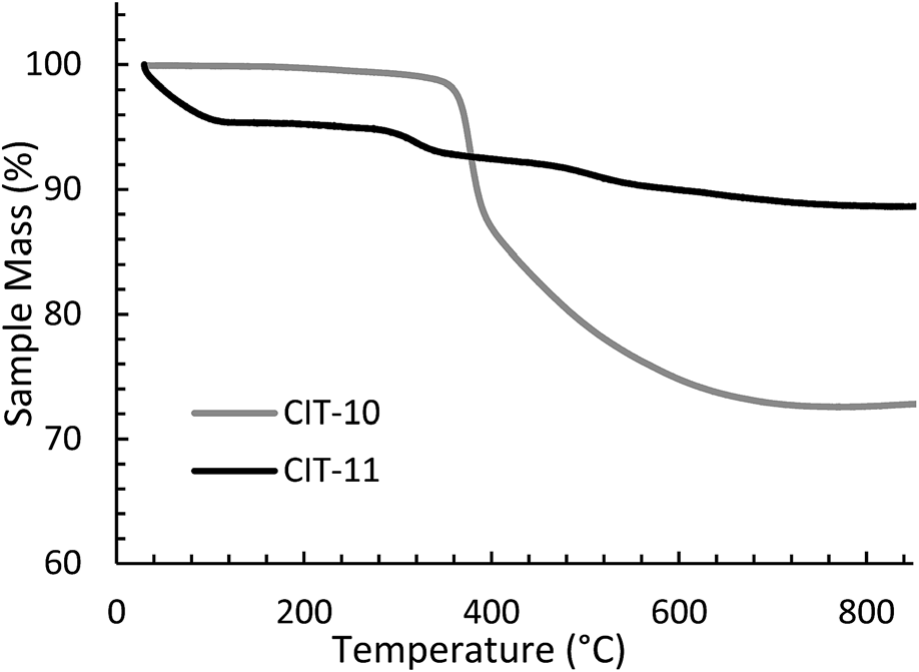
TGA of CIT-10 and CIT-11.

The TGA data are consistent with the condensation temperature observed in the variable temperature PXRD. In the variable temperature PXRD, the structure of the layered material is intact until 330 °C, then the low angle peak corresponding to the 001 direction abruptly disappears. This reflection is absent at the PXRD pattern taken at 380 °C, then begins to emerge around 430 °C. From the TGA trace in Fig. 8, a sharp mass loss occurring around 375 °C is seen, and is in the same temperature range where the low angle peak disappeared in the variable temperature PXRD. The rapid change observed with **RTH** is in contrast to the TGA trace and structural changes observed with CIT-8P (layered precursor to **HEU**) where a gradual shift in position of the low-angle peak was observed along with the gradual decrease in mass.^34^

A detailed analysis of the structural changes occurring during the topotactic condensation process for RUB-39 have been reported as a series interconnected endothermic (OSDA decomposition and condensation of silicate layers) and exothermic (OSDA combustion) processes that are dominated by combustion of the OSDA.^44^ It was found that as soon as the OSDA was removed, the condensation occurred. Attempts to stabilize the intermediate material were not successful. The sharp mass loss over a narrow temperature range observed with CIT-10 is similar to what is reported with RUB-39, and for the latter the loss was ascribed to a fast series of connected processes that include OSDA decomposition and combustion as well as condensation of the silicate layers.

**Fig. 9:**
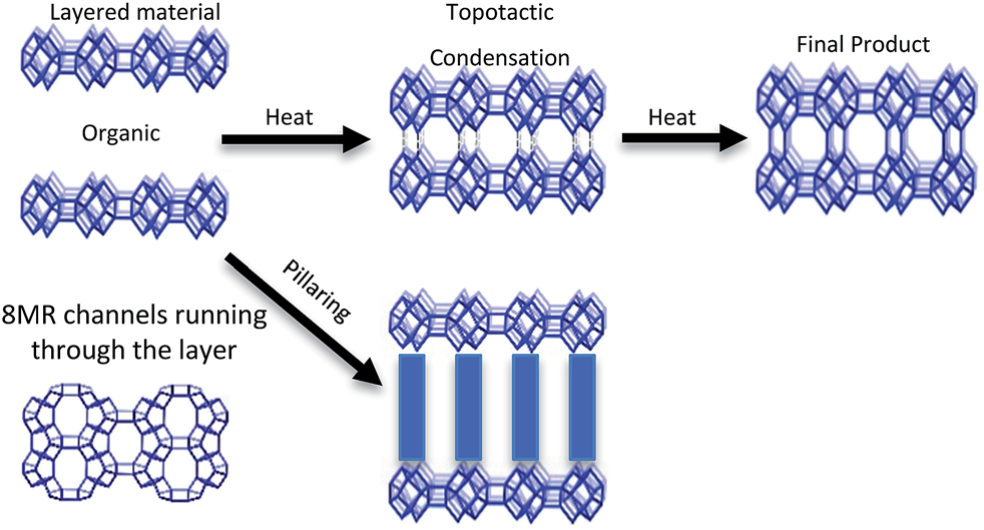
Depiction of the topotactic condensation and pillaring of CIT-10.

CIT-10 has an 8 MR channel running through the layer along the *c* axis, with dimensions of 2.5 × 5.6 Å. As the structure condenses along the *c*-axis, a second 8 MR channel system running through the *a*-axis is formed, and a cavity is created at the intersection of the two 8 MR channels, forming the **RTH** framework structure. The condensation process is shown schematically in Fig. 9. As the schemes in the figure depict, the **RTH** layer is actually a half unit cell thick compared to the final **RTH** framework unit cell.

Table 1 shows the comparisons of the *d*-spacing corresponding to the first and most intense PXRD peak for known 2D layered materials, and the corresponding *d*-spacing shrinkage after topotactic condensation to form 3D framework materials. In most of the cases (including CIT-10) the *d*-spacing shrinkage due to topotactic condensation is around 2 Å, an observation that has been discussed by Roth *et. al.*^46^ It is also interesting to note that although the OSDAs used to make CIT-10 and CIT-8P are very similar, the latter demonstrated a *d*-spacing contraction nearly twice that of the former.

**Table 1:** Comparisons of the *d*-spacing corresponding to the first and most intense PXRD peak for known 2D layered materials, and the corresponding *d*-spacing shrinkage after topotactic condensation to form 3D framework materials

2D zeolite	*d*-spacing (Å)	3D zeolite	Corresponding *d*-spacing (Å)	*d*-spacing shrinkage (Å)	Ref.
CIT-10	11.8	Siliceous **RTH**	9.8	2.0	This work
RUB-36	11.1	RUB-37 (**CDO**)	9.2	1.9	45
MCM-22P	26.9	MCM-22 (**MWW**)	24.9	2.0	16, 46
HMP-2	17.5	MCM-35 (**MTF**)	15.4	2.1	22
RUB-39	10.8	RUB-41 (**RRO**)	8.7	2.1	24
R-RUB-18	9.1	RUB-24 (**RWR**)	6.8	2.3	25
EU-19	11.5	EU-20 (**CAS-NSI**)	8.3	3.2	19
PREFER	13.1	**FER**	9.4	3.7	15
CIT-8P	12.8	CIT-8 (**HEU**)	8.9	3.9	34

### 3.2. Pillaring of CIT-10

In some cases it is possible to pillar layered materials using a monomeric silane in order to prepare materials with pores larger than would have been formed by topotactic condensation. These materials are commonly referred to as interlayer expanded zeolites (IEZ), and they have been prepared from precursors such as PREFER, MWW(P), PLS-1, MCM-47, RUB-36, RUB-39 and Nu-6(1).^8^,^30^,^31^ Pillaring is normally carried out in acidic media, under hydrothermal conditions, and two of the most common pillaring agents are dichlorodimethylsilane and diethoxydimethylsilane.

In attempting to pillar CIT-10, a wide range of conditions were explored including acid type and concentration, aqueous *versus* ethanolic acid, silane source, and reaction temperature and time. The optimal conditions to pillar CIT-10 were found to be 1.25 M HCl in ethanol with either dichlorodimethylsilane or diethoxydimethylsilane at 175 °C for 24 hours. Other conditions lead to what appeared to be pillared materials (based on PXRD), but these materials exhibited very weak X-ray reflections. We interpret these results as indicating framework destruction, and these solids were often not stable to calcination. This phenomenon has been observed before, *i.e.*, that acidic ethanol is the only effective medium to carry out pillaring. It has been postulated that the reason for this is that effective pillaring takes place when the rate of removal of OSDA is well matched by the rate of silylation.^30^ It should be noted that while we made no special efforts to preclude trace amounts of water in these syntheses (such as working in a glovebox or using a Schlenk line), the water content was likely very low.

**Fig. 10:**
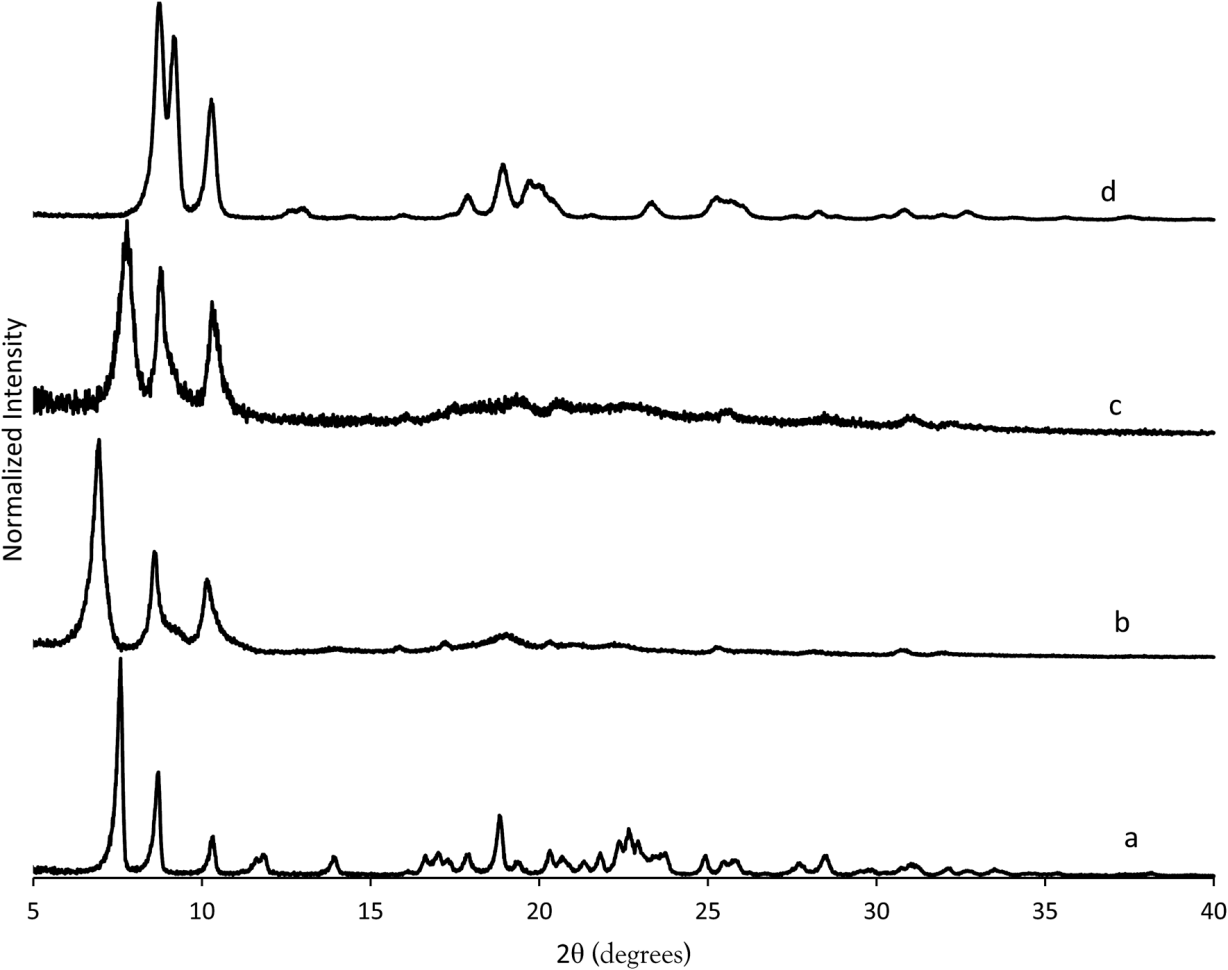
PXRD patterns of (a) CIT-10, (b) CIT-11, (c) CIT-12 and (d) pure-silica **RTH**.

The X-ray diffraction results of pillared CIT-10 are shown in Fig. 10. As can be observed from the PXRD patterns, pillaring causes a shift in the most intense reflection from 7.5° 2*θ* in CIT-10 to 6.8° 2*θ* in CIT-11 (*i.e.*, 1.1 Å expansion). This peak continues to shift to 7.7° 2*θ* in CIT-12 (after calcination). The ^13^C CPMAS NMR of CIT-11 (Fig. 4) shows that the majority of the organic was removed under acidic conditions (while CPMAS NMR is not quantitative this was confirmed by TGA, shown in Fig. 8). This result was expected as a change in color of the acidic medium was observed. The strong resonance observed near –1 ppm is consistent with (CH_3_)_2_Si carbon that is expected from the pillaring. (Prior to NMR analysis it was necessary to degas the material under vacuum at 150 °C to remove any residual ethanol or acetone.) The TGA analysis of CIT-11 (Fig. 8) shows several distinct mass loss regions. The first mass loss of 5% is attributed to the loss of water and possibly residual ethanol or acetone (material was dried in air at 100 °C prior to analysis but not under vacuum as was done with the NMR sample). The second sharp mass loss begins around 300 °C, and is attributed to removal of residual organic (present in ^13^C CPMAS NMR). There is a third, distinct region of mass loss that begins around 500 °C and is attributed to combustion of the Si–CH_3_ groups to form hydroxyl groups.

The ^29^Si NMR spectra are consistent with a pillared material (Fig. 5). In CIT-10, both Q^4^ and Q^3^ silicon environments are observed, consistent with a layered material (*vide supra*). In the pillared material, CIT-11, ^29^Si NMR resonances are observed at –113.5, –108.4, –104.5 and –15.3 ppm with approximate area ratios of 20 : 8 : 2 : 5. The resonances at –113.5 and –108.4 are assigned to Q^4^ silicon and the resonance at –104.5 is assigned to residual Q^3^ silicon. The Q^3^/(Q^3^ + Q^4^) ratio in the pillared material is 0.07, a significant decrease from 0.23 in CIT-10, indicating that a substantial amount of Q^3^ species have been consumed in linking the layers of the material. The resonance at –15.3 is assigned to bridging silanol groups bonded to two methyl groups, that is Si(CH_3_)_2_(OSi)_2_ coordination. The ratio of (Q^2^ + Q^3^)/(Q^2^ + Q^3^ + Q^4^) is 0.25, consistent with the expected value from the **RTH** layer (see ESI†). Upon calcination the material exhibits a broad resonance around –110 ppm. A single broad resonance has been observed in other pillared, calcined materials such as PLS-4.^47^ No obvious peak for Q^2^ silicon can be seen near –90 ppm, but it is likely obscured by the much broader resonance.

The structure of CIT-11/12 is a 3D pore system consisting of 8 and 10 MRs, shown in Fig. 9. The 8 MR running the in the *c*-direction perpendicular to the **RTH** layer remains intact. The pillars form two new ring sizes as the previous 8 MR along the *a*-direction expands to a 10 MR and the 6 MR along the *b*-direction expands to an 8 MR. This means that the previous 2D ring system in **RTH** expands be a 3D ring system in CIT-11/12.

The pore system of CIT-12 was confirmed using argon adsorption. The results from this analysis are shown in Fig. S2† compared to pure-silica **RTH** as well as zeolite 5A and pure-silica **BEA**. We chose to plot all of these isotherms on the same graph as the shape of the isotherm in the low pressure regime is an indication of the pore size distribution. The comparison of the low pressure region of the argon adsorption isotherms indicates that this material has a pore system that has expanded compared to pure-silica **RTH** (8 MRs) but is smaller than pure-silica **BEA** (12 MRs), consistent with the structure solution.

## 4. Conclusions

A new 2D building layer has been synthesized, the rth type layer, denoted CIT-10. This is the first reported 2D building layer that contains small pores that are perpendicular to the layer. Upon calcination, this material forms pure-silica **RTH**, making pure-silica **RTH** accessible without using a difficult to synthesize organic. CIT-10 can be pillared, forming CIT-11, that can then be calcined, forming CIT-12. CIT-12 contains a 3D pore system of 8 and 10 MRs. As CIT-10 contains a new 2D layer there are many further studies to be conducted with this material such as swelling, exfoliation and delamination as well as heteroatom introduction to introduce sites for ion exchange and catalysis. CIT-10 is the first layered material to contain small pores running through the layer; it is possible this material could find use in separations, especially of small molecules. Such possibilities have already been explored with other microporous material frameworks such as **LTA** and **MFI**. However, the **RTH** layers are only half a unit cell thick compared to a full unit cell with **MFI**^27^ (which has medium pores) and multiple unit cells with **LTA**^48^–^50^ (small pores). Additionally, the **RTH** layers have elliptical pores running through them, which may offer additional size discrimination compared to other small pore materials with circular pores, such as **LTA**.

## Supplementary Material

Supplementary information
