# Supplementary material for: Synthesis of the RTH-type layer: the first small-pore, two dimensional layered zeolite precursor
Source: Chem Sci. 2015 Jul 27;6(10):5955–63. doi: 10.1039/c5sc02325d (PMC5950832; doi:10.1039/c5sc02325d)
Supplement: Supplementary file 1 [file SC-006-C5SC02325D-s001.pdf]

Electronic Supplementary Material (ESI) for Chemical Science.
This journal is © The Royal Society of Chemistry 2015

Synthesis of the RTH-Type Layer: The First Small-Pore, Two-Dimensional Layered Zeolite Precursor

Joel E. Schmidt[1], Dan Xie[2], and Mark E. Davis[1]*

[1]Chemical Engineering, California Institute of Technology, Pasadena, CA 91125, USA

[2]Chevron Energy Technology Company, Richmond, CA 94802, USA

*mdavis@cheme.caltech.edu

Supporting Information

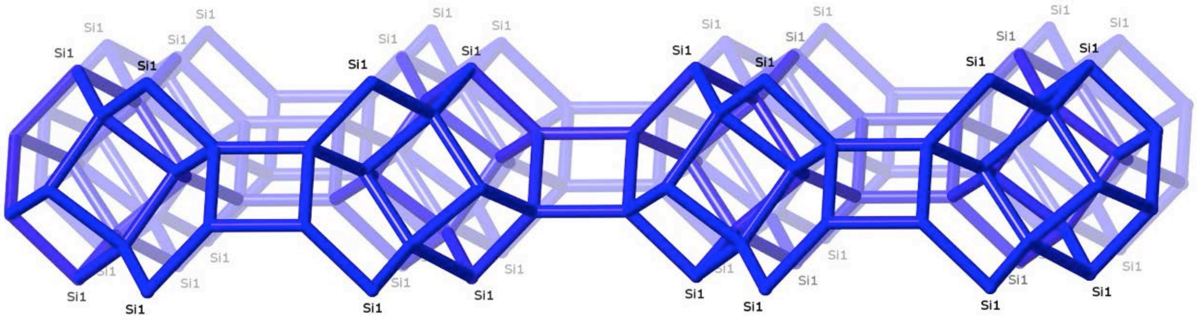

Figure S1. RTH building layer showing *4 independent T atoms*. As all 4 have the same multiplicity and only T-1 atoms are on the surface, *ideally* $Q^3/Q^4 = 1/4 = 0.25$.

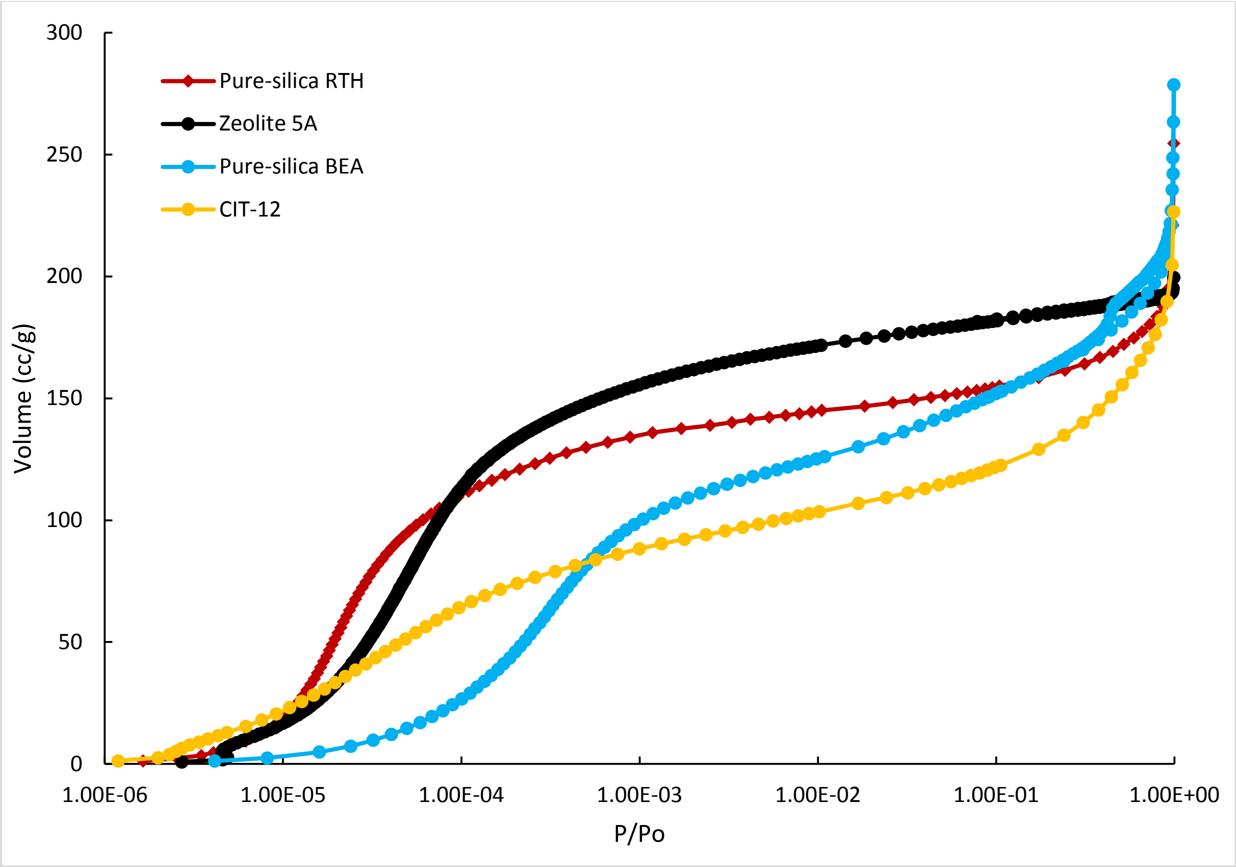

Figure S2. Argon adsorption isotherms.
